# Supplementary material for: Cascade Förster Resonance Energy Transfer Studies for Enhancement of Light Harvesting on Dye-Sensitized Solar Cells
Source: Nanomaterials (Basel). 2022 Nov 20;12(22):4085. doi: 10.3390/nano12224085 (PMC9694053; doi:10.3390/nano12224085)
Supplement: Supplementary file 1 [file nanomaterials-12-04085-s001.zip › nanomaterials-2046295-supplementary.pdf]

nanomaterials

Supporting Information

Cascade Förster Resonance Energy Transfer Studies for Enhancement of Light Harvesting on Dye-Sensitized Solar Cells

Mulugeta Tesema Efa [1,†], Jheng-Chang Huang [2,†] and Toyoko Imae [1,2,*]

1 Graduate Institute of Applied Science and Technology, National Taiwan University of Science and Technology, Taipei 10607, Taiwan
2 Department of Chemical Engineering, National Taiwan University of Science and Technology, Taipei 10607, Taiwan
* Correspondence: imae@mail.ntust.edu.tw
† These authors contributed equally to this work.

Table S1. Evaluated parameters for mixing solutions of Py, AC and AO. (V: volume).

Solution	V_{Py} (mL)	V_{AC} (mL)	V_{AO} (mL)	V_T (mL)	[Py] (μM)	[AC] (mM)	[AO] (mM)	V_{AC} (mL)	V_{AO} (mL)	V_{Py} (mL)	V_T (mL)	[Py] (μM)	[AC] (mM)	[AO] (mM)
Py	5.00	0.00	0.0	5.00	3.00	0.000	0.00	5.00	0.00	0.0	5.00	0.0	3.00	0.000
Py + AC	5.00	0.02	0.0	5.02	2.98	0.011	0.0	5.00	0.02	0.0	5.02	0.0	2.99	0.011
Py + AC	5.00	0.04	0.0	5.04	2.97	0.023	0.0	5.00	0.04	0.0	5.04	0.0	2.98	0.023
Py + AC	5.00	0.06	0.0	5.06	2.96	0.035	0.0	5.00	0.06	0.0	5.06	0.0	2.97	0.035
Py + AC	5.00	0.08	0.0	5.08	2.95	0.047	0.0	5.00	0.08	0.0	5.08	0.0	2.95	0.047
Py + AC	5.00	0.10	0.0	5.10	2.94	0.058	0.0	5.00	0.10	0.0	5.10	0.0	2.94	0.058
Py + AC	5.00	0.16	0.0	5.16	2.90	0.090	0.0	5.00	0.16	0.0	5.16	0.0	2.90	0.090
Py + AC	5.00	0.18	0.0	5.18	2.89	0.100	0.0	5.00	0.18	0.0	5.18	0.0	2.89	0.100
Py + AC	5.00	0.20	0.0	5.20	2.88	0.120	0.0	5.00	0.20	0.0	5.20	0.0	2.88	0.120
Py + AC	5.00	0.22	0.0	5.22	2.87	0.130	0.0	5.00	0.00	0.0	5.22	0.0	2.70	0.13
Py + AC + AO	5.00	0.00	0.00	5.00	3.00	3.000	3.0000	0.0	0.00	5.00	5.00	3.00	0.0	0.000
Py + AC + AO	5.00	0.22	0.02	5.24	2.97	0.125	0.0114	0.0	0.02	5.00	5.02	2.99	0.0	0.011
Py + AC + AO	5.00	0.22	0.04	5.26	2.96	0.125	0.00114	0.0	0.04	5.00	5.04	2.98	0.0	0.023
Py + AC + AO	5.00	0.22	0.06	5.28	2.95	0.125	0.0113	0.0	0.06	5.00	5.06	2.97	0.0	0.035
Py + AC + AO	5.00	0.22	0.08	5.30	2.95	0.124	0.0113	0.0	0.08	5.00	5.08	2.95	0.0	0.047
Py + AC + AO	5.00	0.22	0.10	5.32	2.94	0.124	0.00112	0.0	0.10	5.00	5.10	2.94	0.0	0.058
Py + AC + AO	5.00	0.22	0.16	5.38	2.92	0.122	0.00111	0.0	0.16	5.00	5.16	2.90	0.0	0.090
Py + AC + AO	5.00	0.22	0.18	5.40	2.89	0.122	0.00111	0.0	0.18	5.00	5.18	2.89	0.0	0.100
Py + AC + AO	5.00	0.22	0.20	5.42	2.87	0.121	0.00110	0.0	0.20	5.00	5.20	2.88	0.0	0.120
Py + AC + AO	5.00	0.22	0.22	5.44	2.86	0.121	0.00110	0.0	0.22	5.00	5.22	2.70	0.0	0.130

Table S2. Evaluated parameters for mixed solutions of Py (3.5 μM), AC (4.9 mM) and AO (4.9 mM). (V: volume).

Solution	V_{Py} (mL)	V_{AC} (mL)	V_{AO} (mL)	Φ	Φ_T	r_1 (Å)	r_3 (Å)	k_T (10^{-3}) (ns)	r_1/R_{01}	Φ_{st}/Φ
Py	5.00	0.00	0.00	0.650	0.000	—	—	—	—	1.000
Py + AC	5.00	0.02	0.00	0.501	0.229	53.113	—	0.723	1.225	1.297
Py + AC	5.00	0.04	0.00	0.382	0.412	46.038	—	1.710	1.061	1.699
Py + AC	5.00	0.06	0.00	0.312	0.521	42.780	—	2.650	0.986	2.086
Py + AC	5.00	0.08	0.00	0.239	0.633	39.616	—	4.200	0.913	2.723
Py + AC	5.00	0.10	0.00	0.181	0.722	37.006	—	6.320	0.853	3.593
Py + AC	5.00	0.12	0.00	0.152	0.767	35.572	—	8.020	0.820	4.287
Py + AC	5.00	0.14	0.00	0.112	0.827	33.413	—	11.670	0.770	5.786

Py + AC	5.00	0.16	0.00	0.098	0.849	32.531	—	13.700	0.750	6.619
Py + AC	5.00	0.18	0.00	0.077	0.882	31.033	—	18.180	0.715	8.455
Py + AC*	5.00	0.20	0.00	0.068	0.895	30.323	—	20.890	0.699	9.567
Py + AC + AO	5.00	0.00	0.02	0.064	0.902	29.967	19.718	22.430	0.691	10.195
Py + AC + AO	5.00	0.00	0.04	0.060	0.908	29.615	19.486	24.070	0.683	10.870
Py + AC + AO	5.00	0.00	0.06	0.054	0.916	29.108	19.153	26.700	0.671	11.947
Py + AC + AO	5.00	0.00	0.08	0.051	0.921	28.799	18.949	28.470	0.664	12.673
Py + AC + AO	5.00	0.00	0.10	0.047	0.927	28.373	18.669	31.130	0.654	13.763
Py + AC + AO	5.00	0.00	0.12	0.044	0.932	28.061	18.464	33.270	0.647	14.639
Py + AC + AO	5.00	0.00	0.14	0.040	0.938	27.551	18.128	37.140	0.635	16.226
Py + AC + AO	5.00	0.00	0.16	0.037	0.942	27.226	17.914	39.880	0.628	17.349
Py + AC + AO	5.00	0.00	0.18	0.036	0.945	26.992	17.761	41.990	0.622	18.218
Py + AC + AO*	5.00	0.00	0.20	0.034	0.947	26.789	17.627	43.950	0.618	19.019

Table S3. Evaluated parameters for mixed solutions of Py (5 μM), AC (7 mM) and AO (7 mM). (V: volume).

Solution	V_{Py} (mL)	V_{AC} (mL)	V_{AO} (mL)	Φ	Φ_T	r_1 (Å)	r_3 (Å)	k_T (10^{-3}) (ns)	r_1/R_{01}	Φ_{st}/Φ
Py	5.00	0.00	0.00	0.650	0.000	—	—	—	—	1.000
Py + AC	5.00	0.02	0.00	0.450	0.307	49.669	—	1.080	1.145	1.443
Py + AC	5.00	0.04	0.00	0.342	0.474	44.134	—	2.200	1.018	1.901
Py + AC	5.00	0.06	0.00	0.269	0.564	40.908	—	3.470	0.943	2.421
Py + AC	5.00	0.08	0.00	0.209	0.587	38.290	—	5.150	0.883	3.113
Py + AC	5.00	0.10	0.00	0.152	0.766	35.584	—	8.000	0.820	4.280
Py + AC	5.00	0.12	0.00	0.117	0.820	33.691	—	11.100	0.777	5.553
Py + AC	5.00	0.14	0.00	0.097	0.851	32.431	—	13.960	0.748	6.724
Py + AC	5.00	0.16	0.00	0.075	0.884	30.895	—	18.680	0.712	8.657
Py + AC	5.00	0.18	0.00	0.062	0.905	29.814	—	23.130	0.687	10.482
Py + AC*	5.00	0.20	0.00	0.049	0.925	28.551	—	29.980	0.658	13.294
Py + AC + AO	5.00	0.00	0.02	0.046	0.929	28.281	18.609	31.740	0.652	14.013
Py + AC + AO	5.00	0.00	0.04	0.043	0.935	27.848	18.324	34.820	0.642	15.277
Py + AC + AO	5.00	0.00	0.06	0.039	0.940	27.445	18.058	38.010	0.633	16.584
Py + AC + AO	5.00	0.00	0.08	0.036	0.945	26.999	17.765	41.930	0.622	18.192
Py + AC + AO	5.00	0.00	0.10	0.034	0.948	26.747	17.600	44.350	0.617	19.186
Py + AC + AO	5.00	0.00	0.12	0.032	0.951	26.416	17.381	47.800	0.609	20.597
Py + AC + AO	5.00	0.00	0.14	0.029	0.955	26.084	17.163	51.560	0.601	22.143
Py + AC + AO	5.000	0.000	0.160	0.027	0.959	25.624	16.860	57.380	0.591	24.528
**Py + AC + AO**	5.00	0.00	0.18	0.025	0.962	25.333	16.669	61.450	0.584	26.192
**Py + AC + AO***	5.00	0.00	0.20	0.024	0.962	25.273	16.629	62.330	0.583	26.553

Table S4. Evaluated parameters for mixed solutions of Py (6.5 μM), AC (9.1 mM) and AO (9.1 mM). (V: volume).

Solution	V_{Py} (mL)	V_{AC} (mL)	V_{AO} (mL)	Φ	Φ_T	r_1 (Å)	r_3 (Å)	k_T (10^{-3}) (ns)	r_1/R_{01}	Φ_{st}/Φ
Py	5.00	0.00	0.00	0.650	0.000	—	—	—	—	1.000
Py + AC	5.00	0.02	0.00	0.368	0.434	45.344	—	1.870	1.045	1.766
Py + AC	5.00	0.04	0.00	0.258	0.603	40.449	—	3.710	0.933	2.520
Py + AC	5.00	0.060	0.00	0.190	0.707	37.446	—	5.890	0.863	3.415
Py + AC	5.00	0.08	0.00	0.139	0.786	34.908	—	8.980	0.805	4.680
Py + AC	5.00	0.10	0.00	0.103	0.841	32.844	—	12.940	0.757	6.305
Py + AC	5.00	0.12	0.00	0.081	0.876	31.323	—	17.200	0.722	8.051
Py + AC	5.00	0.14	0.00	0.064	0.902	29.972	—	22.400	0.691	10.185
Py + AC	5.00	0.16	0.00	0.048	0.926	28.484	—	30.410	0.657	13.468
Py + AC	5.00	0.18	0.00	0.040	0.939	27.482	—	37.700	0.634	16.458

Py + AC*	5.00	0.20	0.00	0.031	0.952	26.363	—	48.380	0.608	20.838
Py + AC + AO	5.00	0.00	0.02	0.029	0.956	26.009	17.114	52.470	0.600	22.512
Py + AC + AO	5.00	0.00	0.04	0.028	0.956	25.944	17.071	53.260	0.598	22.836
Py + AC + AO	5.00	0.00	0.06	0.027	0.959	25.698	16.909	56.390	0.592	24.120
Py + AC + AO	5.00	0.00	0.08	0.025	0.961	25.403	16.715	60.440	0.586	25.780
Py + AC + AO	5.00	0.000	0.10	0.023	0.964	25.076	16.499	65.330	0.578	27.785
Py + AC + AO	5.00	0.00	0.12	0.022	0.966	24.771	16.299	70.300	0.571	29.824
Py + AC + AO	5.00	0.00	0.14	0.020	0.969	24.467	16.099	75.710	0.564	32.041
Py + AC + AO	5.00	0.000	0.16	0.019	0.970	24.249	15.956	79.890	0.559	33.750
Py + AC + AO	5.00	0.00	0.18	0.018	0.972	24.071	15.839	83.490	0.555	35.224
Py + AC + AO*	5.00	0.00	0.20	0.018	0.973	23.884	15.715	87.490	0.551	36.873
